# 

**Published:** 1882-10

**Authors:** 


### A Reminder.

Let it be kept in mind by all interested, that the Mad-River Dental Society will meet in Dayton, Ohio, Oct. 24th, at 10 o'clock, A. M. It is desirable that all the former members, so far as possible, be present; and those who have not been members, and are within reach, are earnestly invited to attend and become identified with the Society, and do whatever they may to add to the interest and profit of the meeting. Let not the hope of making a "dollar or two" keep any away. The programme, as given in the last number of the REGISTER, is an inviting one, in which all will doubtless be interested; then let all come.

### Absence.

Duties at the University of Ann Arbor, will require my absence from professional business in Cincinnati, a part of each month from October 1st, 1882, till March 30th, 1883.

I will be at my office

October 14th, A.M. till November 1st, A.M.
November 16th, A.M. till December 2d, P.M.
December 16th, A M. till January 8th, P.M.
January 20th, A.M. till February 5th, P.M.
February 19th, A.M. till March 11th, P.M.
Then absent till March 30th.

Mail may be sent to either Cincinnati or Ann Arbor.

J. TAFT, Dentist,
122 W. 7th Street.

### THE DENTAL REGISTER,

A MONTHLY MAGAZINE DEVOTED TO THE INTERESTS OF THE

**DENTAL PROFESSION.**

*Terms Reduced to* $2.00 *Per Year. Single Copies,* 25 *Cents.*

**EDITED BY PROF. J. TAFT, D.D.S.**

—AND—

***Published by SPENCER & CROCKER, Ohio Dental Depot.***

The volume commences in January and closes in December.

The REGISTER being issued monthly is always fresh, therefore Indispensable to the practitioner who desires to keep posted up with the new inventions and improvements which are daily developed. Neither expense nor effort will be spared to give value and variety to its contents. Articles will be illustrated with well executed engravings whenever they will add to the interest or assist to explanation.

Its extensive circulation and frequency of issue make it one of the BEST DENTAL ADVERTISING MEDIUMS in the United States.

All subscriptions must begin with January or July.

Local or special notices, five lines or less, will be inserted for $3 each insertion; over five lines, 50 cts. per line.

All advertising bills payable in advance, or at furthest, after the issue of the first number containing the advertisement. All transient advertisements to be paid for in advance.

☞CONTRIBUTIONS SOLICITED.

Exclusively Editorial Communications should be addressed to the Editor.

The latest number of the REGISTER may be ordered with or without other goods at any time, and all letters should be addressed to

**SPENCER & CROCKER, Ohio Dental Depot, Cincinnati, Ohio.**

